# Digital empowerment for innovative teaching: application and mediating effects of information technology in physical education teachers’ practices

**DOI:** 10.3389/fspor.2025.1612745

**Published:** 2025-07-02

**Authors:** Bochuan Zhao, Junji Chen, Jiefu Yang, Yuqi Su

**Affiliations:** ^1^ School of Physical Education and Health, Guilin University, Guilin, Guangxi, China; ^2^ Academic Affairs Office, Guilin University, Guilin, Guangxi, China; ^3^ Student Affairs Office, Guilin No. 2 Technical School, Guilin, Guangxi, China

**Keywords:** information technology, innovative teaching, physical education, technology integration, teacher performance

## Abstract

**Introduction:**

As innovative teaching and technology integration become increasingly important in physical education (PE), challenges remain in equipping teachers with necessary skills and resources. This study explores the current situation and relationship between PE teachers’ use of information technology (IT) for innovative teaching.

**Methods:**

A sample of 217 PE teachers completed a questionnaire measuring demographic variables, IT teaching, and innovative teaching performance. Quantitative analyses, including descriptive statistics, *t*-tests, ANOVA, and multiple regression, were conducted using SPSS.

**Results:**

Results showed significant differences in IT teaching based on gender (t = −2.15, *p* < .05) and equipment adequacy (F = 4.24, *p* < .01). Participation in research and advanced education led to higher innovative teaching, especially in evaluation and management (t = 3.14, *p* < .01). IT teaching positively predicted innovative teaching performance, with document software having the strongest impact (β = .28, *p* < .001) on assessment and management. Mediation analysis revealed that teaching methods and content fully mediated the relationship between IT teaching and assessment and management.

**Discussion:**

The findings suggest that IT integration allows PE teachers to create more diverse and innovative learning experiences. However, challenges related to teachers’ IT skills and school technology infrastructure need to be addressed. Implications for teacher training, technology policies, and curriculum design are discussed. Future studies could adopt longitudinal designs and mixed methods to further investigate the dynamic processes and mechanisms underlying technology-enhanced innovation in PE.

## 1 Introduction

The rapid advancement of artificial intelligence (AI) and information technology (IT) has revolutionized various sectors. This transformation has significantly impacted education, particularly teaching and learning processes (1, 2). The effective integration of technology has become an inevitable trend that can greatly enhance student motivation and learning outcomes, particularly in the field of physical education (PE) (3, 4). However, successful IT implementation in PE teaching practices faces several challenges. First, PE teachers require the necessary skills and resources to effectively integrate technology into their instruction. Additionally, new technologies demand innovative teaching approaches to maximize student engagement and performance (5–7).

Regular exercise is crucial for developing healthy habits and preventing diseases, and schools play a vital role in cultivating these habits from childhood through PE (8, 9). Shaping a positive campus sports culture has significant implications for fostering students' lifelong engagement in physical activity and overall well-being (10, 11). Traditional PE teaching methods, often dominated by direct instruction and repetitive drills, may not effectively motivate all students to actively participate and enjoy the benefits of physical activity (12, 13). Innovative teaching approaches, such as integrating cross-disciplinary content, leveraging community resources, and employing student-centered pedagogies, have shown promise in enhancing student engagement, skill development, and positive attitudes toward PE (14–16). The integration of IT into PE has the potential to further transform teaching and learning experiences by providing new tools and resources for instruction, assessment, and classroom management (17–19).

The increasing popularity of computer networks and the substantial improvement of transmission efficiency have transformed the closed learning environment of the past into a more open and flexible one, presenting unprecedented challenges and opportunities for PE teachers to inspire knowledge creation and logical thinking (20, 21). IT, encompassing computer software and hardware, network and communication technologies, and application software development tools, has become an essential medium for managing and processing various forms of information in the digital era (22). The effective integration of IT into PE teaching involves not only the use of technological tools but also the alignment of these tools with pedagogical strategies and content knowledge to optimize learning outcomes, as emphasized by the technological pedagogical content knowledge (TPACK) framework (23, 24).

Previous studies have investigated various aspects of technology integration and innovative teaching in PE. For example, Villalba et al. (2017) examined the obstacles perceived by PE teachers in integrating information and communication technologies (ICT) into their classrooms (25). The study highlighted that despite the potential benefits of ICT integration in PE, teachers face unique challenges due to the subject's specific characteristics, such as the importance of the motor component and limitations on space, time, and training. Gibbone et al. (2010) investigated secondary PE teachers' attitudes and practices regarding technology integration. The study highlighted that although teachers generally had positive attitudes about technology use, their reported technology use was limited (26). Thomas and Stratton (2006) conducted a national audit of ICT use in PE departments across England. The study aimed to investigate the equipment used, staff training received, teachers' attitudes, and teaching approaches when using ICT in PE (27). Moreover, Yaman (2008) surveyed PE teachers in Turkey to examine their educational technology usage levels and attitudes, and found that PE teachers have different abilities and attitudes in educational technology (28). Mohnsen (2012) discussed the growing trend of online PE courses and the potential for technology to promote achievement of national PE standards (29). However, the relationship between IT integration and innovative teaching performance in PE remains underexplored, with conflicting views on whether technology facilitates or hinders pedagogical innovation (30, 31). Some researchers argue that technology can provide new tools and resources to support creative teaching practices, while others caution against the uncritical adoption of technology without considering its alignment with learning objectives and student needs (32, 33). As such, there is a need for more empirical evidence to clarify the complex interplay between technology integration and innovative teaching in PE.

Previous studies have highlighted the importance of IT integration in PE teaching, but few have explored the mechanisms through which specific technological tools influence innovative teaching outcomes. Document software, widely used for planning and organizing teaching content, may exert an indirect influence on teachers' ability to evaluate and manage classroom practices. Document software, as used in this study, refers to word processing and document creation applications such as Microsoft Word, Google Docs, WPS Office, and similar programs that enable teachers to create, edit, and share teaching materials, lesson plans, and assessment documents (34). The TPACK framework points out that the influence of technology on teaching effect is realized through the integration of technology, teaching strategy (teaching method) and subject content (24). This study contributes to the literature by examining the mediating role of teaching methods and content in the relationship between document software use and assessment and management in innovative teaching. Understanding this mechanism provides new insights into how technology can be aligned with pedagogical strategies to enhance teaching outcomes. Drawing from the TPACK framework, we conceptualize IT as an enabler that enhances teaching outcomes through pedagogical transformation, rather than as a mediator between existing pedagogical practices and outcomes.

The significance of this study lies in identifying evidence-based strategies for advancing PE through teacher professional development, technology integration, and strategic resource allocation, enabling educational stakeholders to create more inclusive and effective learning experiences (35–38). Moreover, understanding the factors that influence PE teachers' adoption of IT and innovative practices can help address persistent gaps and inequities in technology access and use, particularly along gender lines and across different school contexts (39, 40). This knowledge can inform targeted interventions and support systems to ensure that all PE teachers have the necessary competencies and resources to effectively integrate technology into their teaching practices and engage in pedagogical innovation. In addition, the findings can inform the development of targeted interventions, such as technology-enhanced instructional strategies and adaptive learning tools, to optimize students' physical literacy and lifelong engagement in physical activity. By bridging the gap between research and practice, this study contributes to the advancement of pedagogical innovation and evidence-based teaching in PE. Therefore, this study aims to address the following research questions: (1) What is the current state of IT integration among PE teachers, and how does it relate to their innovative teaching practices? (2) What demographic factors influence PE teachers' adoption of IT and innovative teaching methods? (3) How does IT teaching, particularly document software use, predict innovative teaching performance? (4) What role do teaching methods and content play in mediating the relationship between IT teaching and assessment/management practices?

## 2 Literature review

### 2.1 Sports IT teaching

The increasing popularity of computer networks and substantial improvement in efficient transmission has fundamentally altered educational paradigms, with IT enabling the shift from teacher-centered to learner-centered approaches in PE. This technological evolution has created new pedagogical possibilities that extend beyond traditional classroom boundaries (11). Several key principles should guide the implementation of IT integration in teaching, including determining the demand for teaching materials, ensuring feasibility within existing school resources, aligning with learning theories, and integrating with original subject content. Teachers' IT literacy plays a crucial role in their ability to evaluate and utilize technology effectively in course activities, ultimately contributing to a more efficient learning environment for students (41–43).

### 2.2 Innovative teaching

Innovation is often used interchangeably with creativity in educational contexts. It refers to the process of applying new ideas or practices to improve teaching performance and student outcomes (44). In the context of teaching, innovation involves the deliberate introduction of new objects, knowledge concepts, and technical methods to enhance the learning experience. Each teacher has their own familiar teaching mode, but with the continuous changes and demands of the modern era, traditional methods may no longer effectively motivate students (45). For PE teachers, this includes reimagining physical activities through technology-enhanced instruction and data-driven performance analysis. Innovative teaching can be categorized into different dimensions such as interactive approaches, organized teaching methods, and reflective practices (46). The implementation of teaching innovation requires teachers to flexibly apply basic teaching principles and strategies, such as creating a favorable class atmosphere, providing abundant learning opportunities, arranging courses properly, maintaining clear learning focus, ensuring solid content, engaging in intelligent dialogue, providing sufficient practice application, offering scaffolding support, teaching learning strategies, and facilitating collaborative learning (47).

### 2.3 Sports IT and innovative teaching

Building on the understanding of IT integration and innovative teaching as separate constructs, the following section explores their combined role in PE teaching. This synthesis is crucial for understanding how technology can serve as a catalyst for pedagogical innovation in PE contexts. The relationship between IT and teaching innovation in PE presents a complex picture. While pre-service teachers generally demonstrate positive attitudes and moderate to high abilities in IT integration (48), a significant gap exists between these attitudes and actual implementation. This gap is shaped by both individual factors (gender, education level, personal motivation, and autonomy) and contextual factors (training experiences, time allocation, professional knowledge, and social feedback) (49, 50). Importantly, when teachers successfully navigate these factors, IT integration shows a positive correlation with innovative teaching ability (51). This suggests that understanding the mechanisms bridging technology use and pedagogical innovation is crucial for enhancing PE teaching effectiveness. However, contrasting perspectives emerge regarding the effectiveness of IT integration in PE, qualitative research reveals more nuanced challenges. These include the risk of “habitual distraction” where students are drawn to off-task activities on digital devices (52), potentially undermining the physical activity focus of PE lessons. This dichotomy suggests that successful integration depends not merely on technology availability but on pedagogical alignment, teacher readiness, and contextual factors.

Building on the TPACK framework introduced earlier, recent empirical evidence suggests that the interplay between technological tools and pedagogical strategies operates through specific mechanisms. Document software, for instance, influences teaching outcomes not directly but through its capacity to enhance instructional design and content organization (25). This indirect pathway distinguishes mere technology use from pedagogically meaningful integration. Multimedia software facilitates the integration of dynamic visual and auditory elements into teaching, enhancing student engagement and understanding. Moreover, media equipment, such as projectors and smart boards, supports real-time demonstration and interaction, fostering collaborative learning environments. However, the extent to which teaching methods and content mediate the relationship between document software and assessment and management in innovative teaching has yet to be fully explored. This study builds on the existing literature by examining this mediating mechanism in the context of PE. The directional relationship warrants clarification: technology's impact on teaching innovation operates through enhanced pedagogical practices, not vice versa. When teachers use document software, it first improves their ability to create and organize teaching materials (teaching methods and content), which subsequently enhances their assessment and management capabilities. This process-based understanding shapes our mediation model.

**Figure 1:**
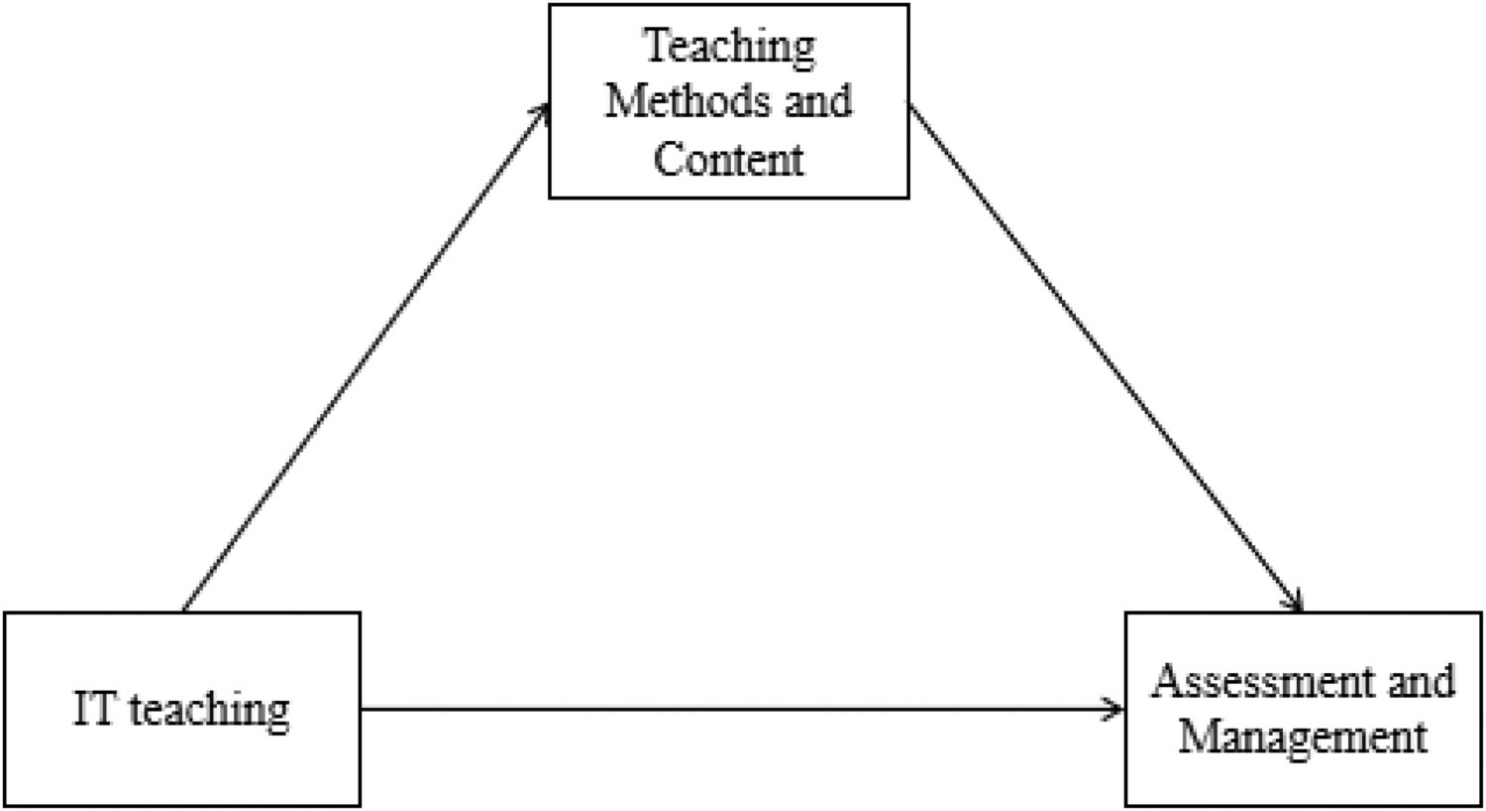
Structural framework.

To address this research gap, the present study examines the current state of IT integration among PE teachers and its impact on their innovative teaching practices. This study is grounded in the TPACK framework, which emphasizes the interplay between teachers' technological knowledge, pedagogical knowledge, and content knowledge in shaping effective technology integration practices. Based on this framework, we propose the following objectives: (1) To examine demographic differences in IT teaching and innovative teaching practices among physical education teachers. (2) To examine the direct and predictive relationships between IT teaching and the two dimensions of innovative teaching (Teaching Methods and Content, and Assessment and Management) among PE teachers; 2) To investigate the mediating role of teaching methods and content in the relationship between IT teaching and assessment and management. Drawing from the identified research gap and previous literature findings, we formulate three hypotheses. First, IT teaching is positively associated with Teaching Methods and Content. Second, IT teaching is positively associated with Assessment and Management (11). Third, Teaching Methods and Content mediates the relationship between IT teaching and Assessment and Management (Figure 1). By considering the multidimensional nature of technology integration and its relationship to pedagogical innovation, this study aims to advance both theory and practice in PE and educational technology.

## 3 Materials and methods

### 3.1 Participants and procedures

The subjects of this study are PE teachers from all public and private schools in Guilin City, Guangxi Province, China. A census of all eligible PE teachers in the city was conducted during the fall semester in 2024. The questionnaires were distributed through Wenjuanxing (http://www.wjx.cn) and school email systems to ensure maximum reach and convenience. This approach was chosen to facilitate access to the target population and increase the response rate, as it allowed teachers to complete the survey at their convenience and reduced the time and cost associated with traditional paper-based surveys. The subjects are requested to fill in the questionnaires carefully. A total of 217 valid responses were received from the 356 teachers contacted, representing a response rate of 61%. This study was approved by the Ethics Committee of Guangxi Normal University (No. 20240705001). Participants were informed that participation was voluntary, and that completing the questionnaire online constituted their informed consent to participate in the study.

### 3.2 Measures

The measurement tool employed in this study consists of adapted scales and an Self-constructed questionnaire for this research: (1) Demographic Variables, (2) Information Technology Integration Teaching Scale, and (3) Innovative Teaching Performance Scale. Each section is designed to collect specific data related to PE teachers' characteristics, their use of IT in teaching, and their innovative teaching performance. The details of each section are as follows:

#### 3.2.1 Demographic variables

Demographic variables of PE teachers: age, gender, teaching experience, highest education, marital status, participation in training and advanced studies, school size and administrative position.

#### 3.2.2 Information technology integrating teaching scale

This study employs the Information Technology Integrating Teaching Scale, which was developed by synthesizing existing literature on technology use in PE education and contextualized for the Chinese PE teaching environment (27, 28). The scale was translated using back-translation procedures and validated through pilot testing with 30 PE teachers. The Information Technology Integration Teaching Scale consists of 26 items, each rated on a 5-point Likert scale. A higher score indicates greater integration of IT in PE instruction, while a lower score suggests less integration of IT in PE teaching. Each item is rated on a 5-point Likert scale (1 = never, 2 = rarely, 3 = sometimes, 4 = often, 5 = always). This scale includes five dimensions: “Multimedia Equipment”, “Word Processing”, “Still Images”, “Video Editing”, and “Internet Resources”. The “Multimedia Equipment” dimension assesses the use of multimedia hardware and software in PE teaching. “Word Processing” evaluates the application of text editing and document creation software. The “Still Images” dimension examines the use of digital images and graphics processing. “Video Editing” assesses the incorporation of video content creation and editing. Lastly, the “Internet Resources” dimension measures the utilization of online materials and resources in PE instruction. For example, sample items include: “I use PowerPoint or similar software to display teaching content in PE lessons” (Multimedia Equipment dimension); “I create lesson plans and teaching materials using word processing software” (Word Processing dimension).” For analysis purposes, these five dimensions were reorganized into three categories based on functional characteristics: (1) Media Equipment (hardware devices); (2) Document Software (text-based tools for lesson planning and materials); and (3) Multimedia Software (combining Still Images, Video Editing, and Internet Resources, as these all involve visual/multimedia content creation and utilization). This structure reflects the practical distinction between hardware infrastructure, document management tools, and multimedia content creation in PE teaching. For the Information Technology Integrating Teaching Scale, the internal consistency coefficients (Cronbach's *α*) for each dimension are as follows: .83 for “Multimedia Equipment”, .82 for “Word Processing”, .89 for “Still Images”, .83 for “Video Editing”, and.82 for “Internet Resources”. According to George and Mallery (2003), Cronbach's *α* values above.8 are considered “good” and above.9 are considered “excellent” (53). All dimensions in both scales demonstrated good to excellent internal consistency.

#### 3.2.3 Innovative teaching performance scale

This research uses an originally developed scale, the “Information Technology Integration in Teaching and Teachers’ Innovative Teaching Performance Questionnaire” to measures the ability to innovate in teaching through the use of technology. This scale consist of 20 items, using a 5-point Likert scale (1 = never, 2 = rarely, 3 = sometimes, 4 = often, 5 = always). Our research team compiled this scale based on existing literature on IT integration in teaching and teachers' innovative teaching performance, as well as interviews with frontline teachers. Specifically, we first identified four dimensions of teachers' innovative teaching performance in an IT environment through a literature review. We then designed five items for each dimension, forming an initial 20-item scale. Finally, a panel of expert PE teachers was consulted to evaluate and revise the scale for clarity and content relevance. A higher score indicates greater innovation in teaching through the use of technology; conversely, a lower score represents less innovation in teaching with technology. This scale includes four dimensions: “teaching method”, “teaching content”, “teaching assessment”, and “class management”. The “Teaching Method” dimension assesses how teachers leverage IT to optimize the teaching process and their motivation. “Teaching Content” dimension assesses how teachers use IT to enrich and update teaching content. “Teaching Assessment” dimension assesses how teachers leverage IT to improve teaching evaluation. “Class Management” dimension assesses how teachers optimize class management with IT. For example, in the “teaching method” dimension, there is an item: “I use multimedia technology to present teaching content in order to improve students’ learning interest.” Another example is an item in the “teaching assessment” dimension: “I use online assessment systems to conduct formative evaluation of students.” Regarding the Innovative Teaching Performance Scale, the internal consistency coefficients (Cronbach's *α*) are: .93 for “Teaching Method”, .87 for “Teaching Content”, .92 for “Teaching Assessment”, and .86 for “Class Management”.

### 3.3 Statistical analysis

The data analysis was conducted using SPSS 22.0. All continuous variables were confirmed with normal distribution by normality measures (skewness and kurtosis) and visual inspection of plots. The statistical methods used in this research include: (1) Descriptive statistics and Pearson correlation analysis to examine relationships among variables; (2) Independent samples *t*-tests and one-way analysis of variance (ANOVA) to compare differences in IT teaching and innovative teaching by demographic variables; (3) Multiple regression analysis was conducted to examine the predictive relationships between IT teaching dimensions and innovative teaching performance. The resulting R^2^ values indicate the proportion of variance explained by the predictors (54); (4) Mediation analysis using PROCESS macro. Moreover, multicollinearity was assessed using variance inflation factors (VIF) and tolerance values for all independent variables. Variables with VIF values less than 10 indicate the absence of multicollinearity. The results showed that the variance inflation factor (VIF) was found acceptable (less than 3). The underlying demographic characteristics were employed as control variables in the mediation analysis.

## 4 Results

### 4.1 Differences between demographic variables and IT teaching

The results of one-way ANOVA and independent samples *t*-test revealed that gender and participation in research and further education showed significant differences in the use of document software and media equipment in IT teaching (see Table 1). These findings are independent of the main structural model and hypotheses but provide valuable context for understanding variations in IT teaching and innovative teaching practices across different groups (Objective 1). Female teachers and those participating in research and professional development showed greater proficiency in using document software or media equipment, suggesting that these factors facilitate more effective technology integration in PE teaching.

**Table 1:** Differences in IT teaching by personal demographic variables.

Variable	Category	Multimedia software			Document software			Media equipment		
		Mean	SD	t/F	Mean	SD	t/F	Mean	SD	t/F
Age	22–30	2.53	.82	.31	3.11	.87	.43	3.03	.97	.12
	31–37	2.63	.71		3.28	.72		3.34	.79	
	38–60	2.73	.88		3.26	.96		3.21	.92	
Gender	Male	2.58	.90	−1.14	3.10	.89	−2.15*	3.16	.96	-.43
	Female	2.70	.69		3.35	.80		3.21	.83	
Teaching experience	≤5 years	2.48	.77	.31	3.09	.79	.51	2.99	.87	.14
	6–10 years	2.68	.79		3.30	.85		3.31	.90	
	11–24 years	2.72	.83		3.26	.89		3.27	.89	
	≥25 years	2.80	1.28		3.14	1.34		2.97	1.31	
Highest education	General University	2.55	.79	.51	3.14	.80	.22	3.10	.87	.24
	Teachers College	2.69	.70		3.11	.73		3.10	.80	
	Master or above	2.68	.89		3.34	.97		3.31	.97	
Marital status	Single	2.55	.76	.31	3.21	.84	.94	3.10	.96	.44
	Married	2.69	.86		3.21	.88		3.26	.86	
	Other	3.15	.21		3.43	.61		3.20	.28	
Participation in research and further education	Yes	2.66	.82	.58	3.31	.92	2.25*	3.32	.91	2.87*
	No	2.59	.80		3.05	.73		2.95	.84	

* *p* < .05.

Moreover, the sufficiency of teaching equipment (e.g., computers, projectors, interactive whiteboards, tablets) had a significant impact on the use of document software and media equipment in IT teaching (Table 2). Interestingly, teachers who reported having sufficient equipment scored lower in these dimensions compared to those with just enough or insufficient equipment. This finding may indicate that teachers with limited resources are more motivated to make the most of the available technology and find innovative ways to integrate it into their teaching practices. Age, teaching experience, highest education, marital status, school size, and administrative position did not show significant differences in any dimensions of IT teaching (Tables 1, 2).

**Table 2:** Differences in IT teaching by school demographic variables.

Variable	Category	Multimedia software			Document software			Media equipment		
		Mean	SD	t/F	Mean	SD	t/F	Mean	SD	t/F
School size	≤24 classes	2.69	.71	.33	3.31	.83	.34	3.34	.80	.61
	25–48 classes	2.73	.70		3.31	.69		3.19	.72	
	≥49 classes	2.56	.89		3.14	.95		3.14	1.02	
Administrative position	Yes	2.60	.85	-.55	3.23	.88	.31	3.27	.90	1.22
	No	2.66	.79		3.20	.85		3.12	.91	
Teaching equipment	Abundant	2.44	.75	1.79	2.94b	.82	3.42*	2.87b	.80	4.24*
	Just enough	2.70	.87		3.29a	.90		3.31a	.95	
	Insufficient	2.67	.71		3.32a	.77		3.19	.82	

Means with different letters (a, b) differ significantly at *p* < .05.

SD, Standard Deviation; t, *t*-test statistic; F, *F*-test statistic.

*p < .05.

### 4.2 Differences between demographic variables and innovative teaching

The results revealed that participation in research and further education was the only background variable that showed a significant difference in innovative teaching, specifically in the assessment and management dimension (Table 3). This finding underscores the importance of ongoing professional development and engagement in research activities for fostering innovative teaching practices among PE teachers. Other demographic variables, including age, gender, teaching experience, highest education, marital status, school size, administrative position, and teaching equipment, did not show significant differences in any dimensions of innovative teaching (Tables 3, 4).

**Table 3:** Differences in innovative teaching by personal demographic variables.

Variable	Category	Teaching methods and content			Assessment and management		
		Mean	SD	t/F	Mean	SD	t/F
Age	22–30	3.48	.57	.30	3.65	.58	.11
	31–37	3.55	.51		3.70	.47	
	38–60	3.55	.72		3.68	.78	
Gender	Male	3.51	.61	-.47	3.63	.64	−1.27
	Female	3.55	.60		3.74	.60	
Teaching experience	≤5 years	3.48	.52	.80	3.67	.56	.14
	6–10 years	3.53	.62		3.66	.57	
	11–24 years	3.54	.63		3.68	.66	
	≥25 years	3.87	1.05		3.83	1.39	
Highest education	General University	3.56	.58	1.31	3.70	.60	2.54
	Teachers College	3.39	.67		3.49	.65	
	Master or above	3.55	.60		3.74	.62	
Marital status	Single	3.47	.53	.85	3.61	.55	.99
	Married	3.57	.67		3.73	.68	
	Other	3.78	.31		3.83	.24	
Participation in research and further education	Yes	3.58	.60	1.80	3.77	0.60	3.14*
	No	3.42	.61		3.50	0.63	

*p < .05.

**Table 4:** Differences in innovative teaching by school demographic variables.

Variable	Category	Teaching methods and content			Assessment and management		
		Mean	SD	F	Mean	SD	F
School size	≤24 classes	3.62	.56	.55	3.78	.51	.58
	25–48 classes	3.54	.59		3.69	.63	
	≥49 classes	3.49	.63		3.64	.64	
Administrative position	Yes	3.59	.54	1.39	3.73	.53	1.22
	No	3.47	.66		3.63	.69	
Teaching equipment	Abundant	3.50	.65	.07	3.68	.68	.00
	Just enough	3.53	.60		3.68	.62	
	Insufficient	3.55	.58		3.68	.59	

### 4.3 Correlation analysis of each variable

From the correlation analysis in Table 5, it can be seen that most of the IT teaching and innovative teaching are significantly correlated. (1) There is a significant positive correlation among the three dimensions of IT teaching; (2) There is a significant positive correlation between the two dimensions of innovative teaching; (3) “Multimedia Software”, “Document Software”, “Media Equipment” in IT teaching and “Teaching Methods and Content”, “Assessment and Management” in innovative teaching all showed a significant positive correlation between medium and high levels.

**Table 5:** Pearson correlation analysis table of each variable (*n* = 217).

Variables	1	2	3	4	5
1 MS	1.00				
2 DS	.70**	1.00			
3 ME	.59**	.70**	1.00		
4 TMC	.52**	.59**	.47**	1.00	
5 AM	.37**	.50**	.37**	.81**	1.00
Mean	2.63	3.21	3.19	3.52	3.68
(SD)	.81	.86	.90	.61	.62

MS, multimedia software; DS, document software; ME, media equipment; TMC, teaching methods and content; AM, assessment and management.

** *p* < .05.

In terms of the average score, for IT teaching of PE teachers, “Multimedia Software” is (M = 2.63 ± .81), “Document Software” is (M = 3.21 ± .86), “Media Equipment” is (M = 3.19 ± .90), among which “Document Software” has the highest score, indicating that when PE teachers use IT in teaching, document software can best meet the needs of teachers. As for the dimension of innovative teaching performance, “Teaching Methods and Content” is (M = 3.52 ± .61), “Assessment and Management” is (M = 3.68 ± .62), and “Assessment and Management” has the highest score, showing that in terms of innovative teaching, the current PE teachers feel that their performance evaluation of students and the way they get along with students in class management are most in line with their own teaching mode.

### 4.4 The impact of IT teaching on innovative teaching

Simultaneous regression analysis was used to explore the influence of IT teaching on innovative teaching (Table 6). The three dimensions of IT teaching, “Multimedia Software”, “Document Software”, and “Media Equipment”, were taken as independent variables, and the two dimensions of innovative teaching, “Teaching Methods and Content” and “Assessment and Management”, were taken as dependent variables. The results show that “Multimedia Software” and “Document Software” have significant predictive power for “Teaching Methods and Content”, with an overall R^2^ of .41. “Document Software” has a significant predictive power for “Assessment and Management”, with an overall R^2^ of .30. This indicates that IT teaching has an influence on innovative teaching, and “Document Software” is an important variable.

**Table 6:** Regression analysis of IT teaching on innovative teaching.

Variable	Teaching methods and content	Assessment and management
	*β*	β
Multimedia software	.104	-.007
Document software	.256***	.278***
Media equipment	.112*	.122
R^2^	.407	.300
Adjusted R^2^	.398	.290
F	48.650***	30.46***

* *p* < .05; ^**^p < .01; ^***^p < .001.

### 4.5 The mediating role of teaching methods in the impact of IT teaching

**Figure 2:**
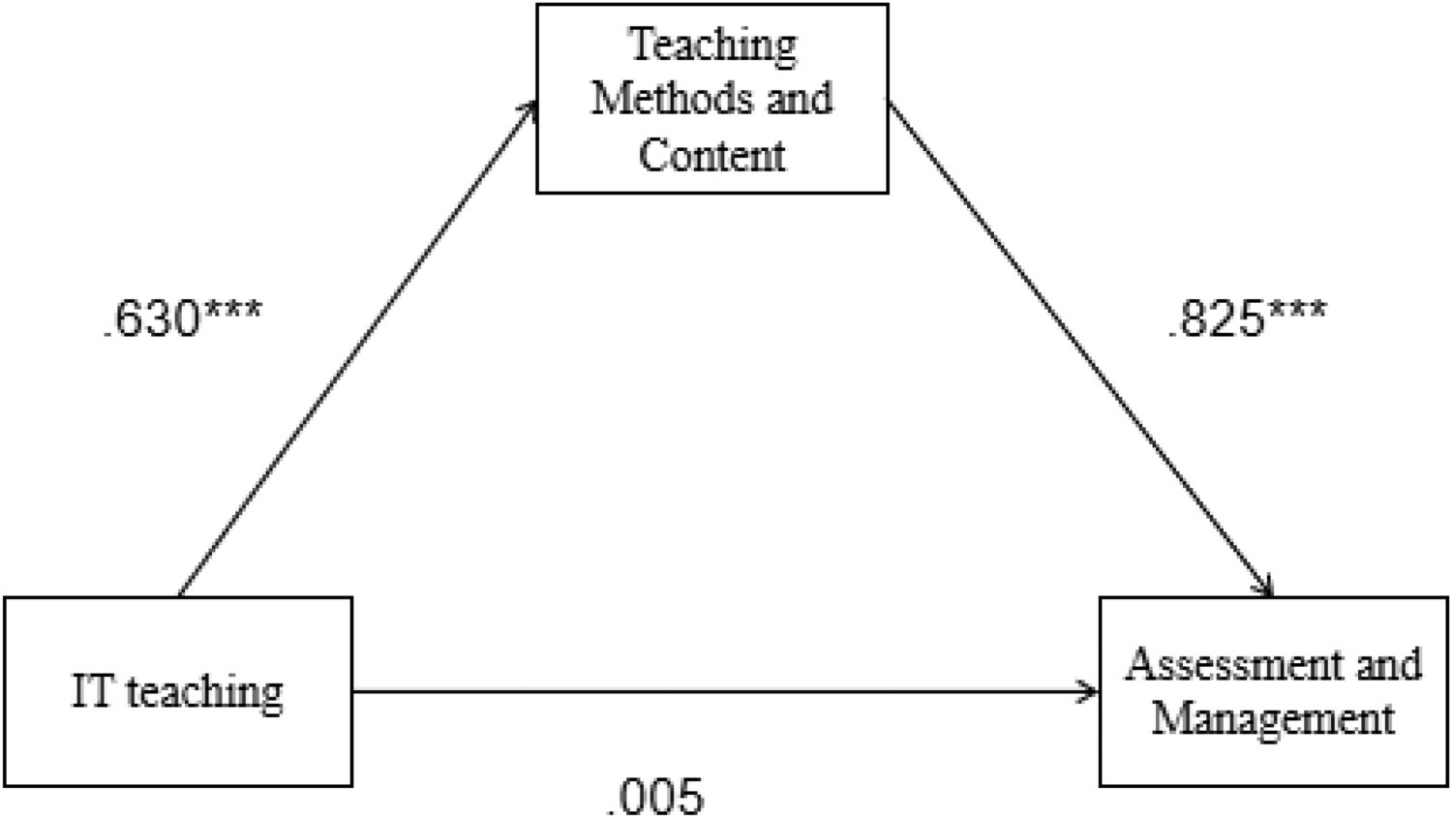
Mediation effect analysis of teaching methods and content between document software and assessment and management; *** *p* < .001.

Mediation analysis based on 5,000 bootstrap samples was conducted to estimate the indirect effects of IT teaching on assessment and management mediated by teaching methods and content. Table 7 illustrates the results of the mediation analysis. The direct effect of IT teaching on assessment and management was not significant (Effect = 0.005, 95% CI: −0.093, 0.103), but the indirect effect was significant (Effect = 0.519, 95% CI: 0.418–0.629). The total effect of IT teaching on assessment and management via the mediation of teaching methods and content was 0.525 (95% CI: 0.411–0.639) (Table 8, Figure 2). The results suggested that teaching methods and content fully mediate the relationship between IT teaching and assessment and management.

**Table 7:** Regression coefficients of the mediating of teaching methods and content between IT teaching and assessment and management.

Outcome variables	Predictor variable	Goodness-of-fit indices			Regression coefficient and significance	
		R	R^2^	F	*β*	t
Assessment and management	IT teaching	.538	.289	28.856***		
					.525	9.069***
Teaching methods and content	IT teaching	.643	.341	49.987***		
					.630	11.987***
Assessment and management	IT teaching	.829	.688	116.824***	.005	.105
	Teaching methods and content				.825	16.462***

*** *p* < .001, ^**^p < .01.

**Table 8:** Mediating effects of teaching methods and content between IT teaching and assessment and management by process. Demographic variables as covariance.

Effect types	Path	95% CI	Effect
Direct effect	IT teaching→Assessment and Management	-.093−.103	.005
Indirect effect	IT Teaching→Teaching Methods and Content→Assessment and Management	.418−.629	.519
Total effect	-	.411−.639	.525

## 5 Discussion

### 5.1 Overview of key findings

This study explored the relationship between IT teaching and innovative teaching among PE teachers in China, as well as the differences in these two aspects based on various demographic variables. The findings provide valuable insights into the current state of IT integration and innovative teaching practices in PE.

### 5.2 IT integration and innovative teaching performance

The analysis of correlation revealed significant positive correlations between the dimensions of IT teaching (multimedia software, document software, and media equipment) and innovative teaching (teaching method and content, assessment and management), which was consistent with hypothesis 1 and 2. However, the strength of these correlations (ranging from.37 to.59) suggests a moderate rather than strong relationship, indicating that technology adoption alone does not guarantee innovative teaching. This finding points to a more detailed understanding: technology is an enabling factor rather than a determining one for teaching innovation. These results align with Juniu's (2011) TPACK framework for technology integration in PE, which posits that effective teaching requires the integration of technological tools (including multimedia software, document processing, and media equipment) with pedagogical strategies and content. Our empirical findings provide quantitative support for this theoretical framework by demonstrating significant positive correlations between these IT dimensions and innovative teaching practices (55). The integration of IT in PE has been shown to enhance students' learning motivation, concentration, self-confidence, and performance of motor skills (56). Therefore, it is crucial for PE teachers to effectively incorporate IT into their teaching practices to create a more engaging and effective learning environment for their students.

The study also found that document software had the highest mean score among the IT teaching dimensions, indicating that it is the most widely used and beneficial tool for PE teachers. This finding is consistent with Woods et al. (2008), who found that 80.7% of PE teachers reported proficiency in using word processors. The high proficiency rates suggest that PE teachers have widely adopted this technology, potentially enabling the diversification of teaching materials and methods observed in our study (57). Document software allows teachers to create, edit, and share various types of documents, such as lesson plans, instructional materials, and assessment rubrics, which can greatly facilitate the planning, implementation, and evaluation of their teaching activities. Moreover, the use of document software can help teachers to organize and manage their teaching resources more efficiently, saving time and effort in the process. The high mean score for the “assessment and management” dimension of innovative teaching suggests that PE teachers perceive their performance evaluation and classroom management methods as most in line with their innovative teaching practices. This finding highlights the importance of assessment and management in innovative teaching, as they provide teachers with the necessary tools and strategies to monitor student progress, provide feedback, and maintain a positive learning environment (58). However, the lower adoption of multimedia software (M = 2.63), despite its potential for dynamic content creation, suggests barriers beyond mere access. This may indicate that multimedia tools require more specialized skills, time investment, or pedagogical redesign that teachers find challenging to implement. Moreover, Media equipment's intermediate position (M = 3.19) is particularly interesting—while readily available in most schools, its impact on innovative teaching was limited (*β*=.112, *p* < .05 for TMC; *β*=.122, n.s. for AM). This suggests that having equipment is necessary but not sufficient for innovation; teachers may use projectors and smart boards for traditional content delivery without leveraging their interactive capabilities.

### 5.3 Demographic influences on IT teaching

Regarding the differences in IT teaching and innovative teaching based on demographic variables, the study revealed some notable findings. Gender and participation in research and further education were found to have significant effects on the use of document software and media equipment in IT teaching. The higher scores among female teachers in document software and media equipment use suggest gender differences in technology adoption patterns (59). However, our finding of higher scores among female teachers contrasts with Teo et al. (2015), who found female pre-service teachers perceived technology use as more challenging (60). This difference reflect the specific educational context of document software and media equipment, where female teachers have developed particular expertise through teaching practice. Female teachers may have developed more positive attitudes and higher self-efficacy in using these specific technologies through their education, training, or personal experiences. Additionally, the nature of the subject matter and the types of technologies used in PE may align more closely with female teachers' pedagogical approaches and preferences. The impact of research and further education on IT teaching underscores the importance of ongoing professional development for teachers to keep abreast of the latest technological developments and pedagogical approaches in their field.

Furthermore, the study found that the sufficiency of teaching equipment had a significant impact on the use of document software and media equipment in IT teaching. Alkasasbeh and Amawi (2024) emphasize that in order to effectively use technology for innovative teaching, PE teacher education programs must provide pre-service teachers with the necessary tools and support to enable them to create technology-enhanced learning experiences. This view is consistent with the significant impact of document software on innovative teaching found in this study, highlighting the critical role of teacher preparation and technological tools in driving innovation in teaching (11). Interestingly, teachers who reported having sufficient equipment scored lower in these dimensions compared to those with just enough or insufficient equipment. This finding aligns with research showing that resource scarcity can enhance creativity by activating a constraint mindset that reduces functional fixedness, leading individuals to think beyond traditional uses and find more innovative solutions (61). This interpretation is supported by previous research that has highlighted the potential for resource constraints to stimulate creativity and innovation in teaching. When faced with limited resources, teachers may be more likely to explore alternative teaching methods and tools, such as open educational resources or free software, to overcome the challenges and meet the learning needs of their students (62). On the other hand, teachers with abundant resources may become complacent and less motivated to seek out new and innovative ways of teaching.

In terms of innovative teaching, participation in research and further education was the only demographic variable that showed a significant difference, specifically in the evaluation and management dimension. This finding underscores the importance of ongoing professional development and engagement in research activities for fostering innovative teaching practices among PE teachers. Previous studies have also emphasized the role of professional development in promoting teacher innovation and enhancing the quality of teaching and learning. Makopoulou and Armour (2014) found that while some teachers were satisfied with exchanging technical advice to solve immediate problems, others sought deeper engagement that challenged their pedagogical assumptions and practices. Their study revealed that meaningful professional learning required not only access to colleagues' practical knowledge but also opportunities to engage with theoretical frameworks and research evidence. Teachers who valued what the authors termed “'theory-practice alchemy'” were more likely to pursue professional development opportunities beyond their immediate school contexts, recognizing that such engagement could transform their understanding and practice (63). Participation in research and further education can expose teachers to new ideas, best practices, and emerging trends in their field, which can inspire them to experiment with innovative teaching approaches and strategies. Moreover, engaging in research can help teachers to develop a more reflective and evidence-based approach to their teaching practice, which can lead to continuous improvement and innovation (64). AlKasasbeh and Amawi's (2024) research also highlights the crucial role of teacher professional development in driving innovation in PE. They recommend designing tailored programs to enhance PE teachers' technological literacy, which aligns closely with the findings and recommendations of the present study. Continuous professional learning and research engagement not only help teachers master cutting-edge teaching concepts and technologies but also cultivate their abilities in critical reflection and evidence-based practice, thereby stimulating the intrinsic motivation for teaching innovation. Future teacher training and development programs should focus on enhancing technology integration capabilities, empowering educational change (11).

### 5.4 Predictive analysis and mediating mechanisms

The regression analysis revealed that multimedia software and document software had significant predictive power for teaching methods and content, while document software alone had a significant impact on assessment and management in innovative teaching. For Teaching Methods and Content, only document software (*β*=.256, *p* < .001) and media equipment (*β*=.112, *p* < .05) showed significant impacts, while multimedia software's effect was non-significant (*β*=.104, n.s.). For Assessment and Management, only document software demonstrated significant predictive power (*β*=.278, *p* < .001), with both multimedia software (*β*=-.007, n.s.) and media equipment (*β*=.122, n.s.) showing no significant effects. This pattern reveals document software's unique versatility across both pedagogical dimensions, while multimedia and media equipment show limited and context-specific impacts. The widespread availability of media equipment contrasted with its minimal predictive power exemplifies technology presence without meaningful pedagogical integration. These findings further support the idea that the use of IT, particularly document software, can facilitate the development and implementation of innovative teaching practices in PE. Multimedia software, such as video editing tools and presentation software, can enable teachers to create more engaging and interactive learning experiences for their students, by incorporating visual and auditory elements into their teaching (24). Document software, as previously discussed, can help teachers to create, organize, and share a wide range of instructional materials and assessment tools, which can support innovative teaching practices. This finding also supports previous research. AlKasasbeh and Amawi (2024) proposed that when integrating technology in PE teacher education, the function of technology as a teaching tool should be emphasized, rather than merely as an aid to classroom management (11). They argue that technology should be conceptualized based on its pedagogical function rather than its specific type or device, shifting the focus towards promoting inquiry, supporting teacher decision-making, and fostering student-centered pedagogy.

The results of the mediation analysis indicate that teaching methods and content play a crucial role in bridging the relationship between IT teaching and assessment and management in innovative teaching, thereby supporting hypothesis 3 (Teaching methods and content mediate the relationship between IT teaching and assessment/management). This complete mediation effect (indirect effect = 0.519, direct effect = 0.005, n.s.) reveals a critical insight: technology's impact on innovative assessment practices is entirely contingent upon its integration with pedagogical methods. This finding problematizes simplistic “technology integration” approaches that focus on tool adoption without corresponding pedagogical transformation. The absence of a direct effect suggests that merely providing teachers with technology access—a common policy approach—is insufficient for improving assessment and management practices. Instead, the mediating mechanism underscores the primacy of pedagogical knowledge in determining whether and how technology enhances teaching innovation, supporting the TPACK framework's emphasis on the intersection rather than addition of technological and pedagogical knowledge. This finding supports Fullan's (2013) Stratosphere framework, which emphasizes that pedagogy must inform technology use rather than technology driving instruction. The mediating role of teaching methods confirms that effective technology integration requires deliberate pedagogical strategies to transform tools into meaningful teaching outcomes (62). It reveals that the use of document software can indirectly enhance teachers' performance in assessment and management by first improving their teaching methods and content. The explanation for this result may be that teachers can use documentation software to create richer teaching materials (such as course Outlines, sample questions, workbooks) and adjust lesson plans in real time based on student feedback (65). Optimized teaching content and methods can help students better understand knowledge points, and systematic teaching design can make classroom management more organized and reduce students' confusion and uncertainty. Moreover, multimedia software significantly influenced teaching methods and content, supporting its role in enhancing student engagement through dynamic and interactive instructional materials. Tools like video editing software allow PE teachers to visually demonstrate techniques and strategies, making lessons more accessible and engaging. Media equipment, though less influential than document software, contributes to innovative teaching by supporting interactive and collaborative activities in the classroom. Tools like smart boards and projectors enable teachers to present content effectively and facilitate real-time student participation. These findings underscore the need for teacher training programs to emphasize not only the use of technology but also its alignment with effective pedagogical practices (66).

### 5.5 Limitations and future directions

However, it is important to acknowledge the limitations of this study. First, the sample was limited to PE teachers in Guilin City, which may limit the generalizability of the findings to other regions or educational contexts. Future research could explore these relationships in a broader sample of teachers across different geographic areas and subject domains. Second, the study relied on self-reported data, which may be subject to response bias. Future studies could employ multiple data collection methods, such as observations and interviews, to triangulate the findings and provide a more comprehensive understanding of the phenomenon (67). Third, this study focused solely on teachers' perspectives and practices without examining the actual impact of IT integration and innovative teaching on student learning outcomes. Future research should incorporate student-level data, including academic performance, physical fitness improvements, motivation levels, and engagement metrics, to provide a more comprehensive understanding of how technology-enhanced innovative teaching actually benefits learners in PE contexts.

### 5.6 Implications and recommendations

Despite these limitations, this study makes a valuable contribution to the literature on IT integration and innovative teaching in PE. The findings provide a foundation for future research and can inform the development of targeted interventions and support systems to enhance the quality of PE teaching in China and beyond. For policymakers, priority should be given to funding document software training programs, as our regression analysis revealed this had the strongest impact on innovative teaching practices (*β*=.256, *p* < .001). Investments in helping teachers master document creation and management tools could significantly enhance pedagogical innovation through improved curriculum design, teaching materials development, and systematic assessment creation. School administrators must address the identified gender gap in IT adoption, with male teachers showing significantly lower document software use than female colleagues (t = −2.15, *p* < .05). Targeted interventions could include peer mentoring programs, collaborative learning environments, and professional development designed to accommodate diverse comfort levels with technology. Creating supportive structures that reduce technology-related anxiety while building confidence is essential for equitable IT integration. For teacher education programs, our mediation analysis demonstrates that technology training must be integrated with pedagogical content knowledge development, as teaching methods and content fully mediate the relationship between IT use and innovative assessment/management practices. Pre-service teachers need to understand not merely how to use technology, but how to use it purposefully to enhance student learning. This requires developing critical thinking skills to evaluate and select appropriate technological tools based on specific pedagogical objectives rather than treating technology as an isolated skill set (68). Our findings align with the TPACK framework by demonstrating that technology (particularly document software) influences teaching effectiveness not directly, but through its integration with pedagogical content knowledge. The mediation effect we observed empirically validates TPACK's proposition that technology, pedagogy, and content knowledge must work synergistically.

## 6 Conclusion

In conclusion, this study provides empirical evidence for the relationship between teaching and innovative teaching among PE teachers in China. The findings highlight the importance of integrating information technology, especially document software, into PE teaching to promote innovative practices and improve teaching effectiveness. By harnessing the potential of technology, PE teachers can create more engaging and interactive learning experiences that enhance student motivation, participation, and ultimately, educational outcomes. The study also identifies several demographic variables, such as gender, participation in research and further education, and the sufficiency of teaching equipment, that may influence the adoption and use of information technology in teaching. Furthermore, this study highlights the mediating role of teaching methods and content in the relationship between IT teaching and assessment and management, suggesting that innovative teaching outcomes depend not only on the use of technology but also on its integration with effective teaching strategies (50). These results have practical implications for education policymakers, school administrators, and teacher training programs, as they can inform efforts to support and enhance the integration of IT and innovative teaching practices in PE. Policymakers and educators should consider leveraging these findings to design targeted interventions and support systems that enhance IT infrastructure, encourage teachers' professional development, and promote the effective integration of technology into PE teaching practices. However, the limitations of this study, such as the reliance on self-reported data and the limited sample size and geographic scope, should be acknowledged. Future research should explore the developmental trajectories of PE teachers' technology integration competencies, identifying critical transition points, support mechanisms, and professional learning designs that facilitate the progression from basic IT use to transformative innovative teaching practices.

## Data Availability

The raw data supporting the conclusions of this article will be made available by the authors, without undue reservation.
